# DAP1 regulates osteoblast autophagy via the ATG16L1–LC3 axis in Graves’ disease-induced osteoporosis

**DOI:** 10.1186/s13018-023-04171-z

**Published:** 2023-09-21

**Authors:** Mingdong Gao, Zouxi Du, Qianqian Dong, Shan Su, Limin Tian

**Affiliations:** 1 The First School of Clinical Medical, Lanzhou University, Lanzhou, 730030 Gansu China; 2 Department Pediatrics, Gansu Provincial Hospital, Lanzhou, 730030 Gansu China; 3 Clinical Research Center for Metabolic Diseases, Lanzhou, 730030 Gansu China; 4 Department of Endocrinology, Gansu Provincial Hospital, No. 204 West Donggang Road, Lanzhou, 730030 Gansu China

**Keywords:** Autophagy, Death-associated protein 1, Graves’ disease, Osteoblasts, Osteoporosis

## Abstract

**Objective:**

This study aimed to uncover a critical protein and its mechanisms in modulating autophagy in Graves’ disease (GD)-induced osteoporosis (OP).

**Methods:**

We discovered the target protein, death-associated protein 1 (DAP1), using bone proteomics analysis. Furthermore, genetic overexpression and knockdown (KD) of DAP1 in bone and MC3T3-E1 cells revealed DAP1 effects on autophagy and osteogenic markers, and autophagic vacuoles in cells were detected using transmission electron microscopy and the microtubule-associated protein 1 light chain 3 alpha (MAP1LC3/LC3) dual fluorescence system. An autophagy polymerase chain reaction (PCR) array kit was used to identify the key molecules associated with DAP1-regulated autophagy.

**Results:**

DAP1 levels were significantly higher in the bone tissue of GD mice and MC3T3-E1 cells treated with triiodothyronine (T3). DAP1 overexpression reduced LC3 lipidation, autophagic vacuoles, RUNX family transcription factor 2 (RUNX2), and osteocalcin (OCN) expression in MC3T3-E1 cells, whereas DAP1 KD reversed these changes. In vivo experiments revealed that GD mice with DAP1 KD had greater bone mass than control mice. DAP1-overexpressing (OE) cells had lower levels of phosphorylated autophagy-related 16-like 1 (ATG16L1) and LC3 lipidation, whereas DAP1-KD cells had higher levels.

**Conclusions:**

DAP1 was found to be a critical regulator of autophagy homeostasis in GD mouse bone tissue and T3-treated osteoblasts because it negatively regulated autophagy and osteogenesis in osteoblasts via the ATG16L1–LC3 axis.

**Supplementary Information:**

The online version contains supplementary material available at 10.1186/s13018-023-04171-z.

## Introduction

Macroautophagy (also known as autophagy) is a dynamic and highly conserved self-eating mechanism that is significantly enhanced in cells exposed to a variety of stressors [1]. It engulfs damaged cellular components or extra metabolites in autophagosomes and fuses them with lysosomes [2], releasing nutrients and energy for cellular recycling [3]. Multiple key molecules strictly execute and regulate the cellular autophagy process. Phosphorylation of the Ser14 site of Beclin-1 by unc-51-like kinase 1 (ULK1), which is activated by autophagy-inducing factors, promotes the activity of the VPS34 complex, thereby inducing cellular autophagy. Thus, phosphorylation of Beclin-1 is necessary for the adequate induction of autophagy in mammals [4]. Microtubule-associated protein1 light chain 3 (MAP1LC3) consists of two interconvertible forms, LC3-I and LC3-II. After induction of autophagy, LC3-I couples to the substrate phosphatidylethanolamine (PE) on the surface of autophagosome membranes by the action of the ATG5-ATG12-ATG16L complex to form a membrane bound form, LC3-II, which is involved in the formation of autophagosome membrane [5]. As a bridge linking LC3 and polyubiquitinated proteins, SQSTM1/p62 (p62) is selectively wrapped into autophagosomes, after which it is degraded by proteolytic hydrolases in autophagic lysosomes, so that the expression of p62 protein is negatively correlated with autophagic activity [6]. Nonetheless, insufficient or excessive autophagy can cause cellular damage and is involved in the pathogenesis of many complex human diseases [1, 7, 8]. Autophagy is critical for bone homeostasis as it regulates the survival, function, and stress response of osteoblasts, osteoclasts, and osteocytes. Autophagy dysfunction, on the other hand, can disrupt bone remodeling homeostasis, inducing or exacerbating osteoporosis (OP) [9, 10]. Nollet et al. reported that autophagy dysfunction in osteoblasts reduced the mineralization capacity and promoted osteoclastogenesis through oxidative stress and overproduction of receptor activator of NF-κB ligand (RANKL). Notably, the trabecular bone volume was reduced by 50% in osteoblast-specific autophagy-deficient mice [11]. As a result, autophagy in osteoblasts contributes to mineralization and bone homeostasis.

Graves’ disease (GD) is a prevalent form of hyperthyroidism that is highly susceptible to secondary OP [12, 13]. The thyroid hormone is critical for bone development as it controls the proliferation and differentiation of chondrocytes, osteoblasts, and osteoclasts [14]. An important factor in GD-induced OP is the imbalance between bone formation and resorption caused by supraphysiologic thyroid hormone doses [15, 16]. Furthermore, triiodothyronine (T3) stimulates osteoblast osteogenesis by activating autophagy [17], consistent with the discovery that rapamycin (Rap), an autophagy agonist, promotes the formation of mineralized nodules in MC3T3-E1 cells [18]. However, the autophagic changes in bone tissue associated with GD and the mechanism of T3-mediated autophagic regulation of osteoblasts remain unknown. As a result, the goal of this study was to see if there are any related proteins that influence osteoblast development via autophagy regulation and thus play a role in GD-induced OP.

We identified the death-associated protein 1 (DAP1) using bone proteomics analysis [19]. DAP1 is an evolutionarily conserved protein that is widely expressed in most eukaryotic cells and tissues [20], negatively regulates cellular autophagy [21], and is closely linked to cell survival, proliferation, and autophagic death [20, 22, 23]. Furthermore, targeting validation revealed that DAP1 levels were significantly higher in the bone tissue of GD mice and T3-treated osteoblasts. The effect of DAP1 on autophagy and osteogenesis was studied in vitro and in vivo using genetic intervention techniques. The underlying molecular mechanism by which DAP1 controls autophagy in osteoblasts was determined using autophagy polymerase chain reaction (PCR) array analysis. The autophagic regulation in bone tissue with GD and T3-treated osteoblasts is complex; DAP1 was found to play a crucial role in the autophagic regulation. It has been demonstrated that regulating cellular autophagy has therapeutic potential for OP [9, 10]. As a result, DAP1 may be a potential therapeutic target for GD-induced OP.

## Materials and methods

### Animal models

Six-week-old female BALB/c mice were obtained from the Laboratory Animal Center of Lanzhou University. They were housed in a 24 °C and 60% humidity-controlled environment with a 12-h light–dark cycle and given free access to food and water. The mice were randomly divided into three groups—GD, negative control (NC), and control groups, and were intramuscularly injected with 1 × 10^9^ plaque-forming units (pfu) of adenovirus carrying the A-subunit of the thyrotropin receptor gene (Ad-TSHR289) (GeneChem, Shanghai, China), a negative virus, and an equal volume of phosphate buffer saline (PBS) for seven cycles, respectively (Additional file 1: Fig. S1A) [24, 25].

Mouse bone DAP1 knockdown (KD) was generated in BALB/c female mice utilizing the AAV9-U6-Dap1-shRNA-CMV-Fluc purchased from GeneChem (Shanghai, China). The number of viral particles in mice in the DAP1-KD groups was 1 × 10^12^ vector genomes (v.g.)/body diluted with PBS to 200 µL; 100 µL was injected into the tail vein, and 100 µL was administered on the periphery of the bilateral femur [26, 27]. The mice in the NC group were inoculated with a similar amount of empty virus in an identical manner. After 2 weeks, the effect of viral infection was examined using a small animal live imaging system (IVIS Lumina III, Connecticut, USA) following the intraperitoneal injection of 10 µL/g of D-fluorescein (Yeasen, Shanghai, China) for 20 min [28]. Immunofluorescence and western blotting were used to verify the effect of DAP1-KD on mouse bone. Animal experimental procedures were performed in accordance with the ARRIVE 2.0 guidelines [29]. All animal experiments were approved by the Ethics Committee of Gansu Provincial Hospital (No. 2022-013).

### Thyroid function test

Following the immunization cycle, serum TSH and TSH receptor antibody (TRAb) were measured using an enzyme-linked immunosorbent assay kit (MEIMIAN, Jiangsu, China), and serum total thyroxine (TT4) was measured using a radioimmunoassay kit (North Institute of Biotechnology, Beijing, China).

### Micro‑computed tomography (micro-CT)

The micro-CT (NEMO II PINGSHENG Healthcare, Shanghai, China) was used to reconstruct the three-dimensional (3D) images of the distal femoral cancellous bones, and microarchitecture parameters such as trabecular volume fraction (Tb.BV/TV), trabecular thickness (Tb.Th), trabecular number (Tb.N), trabecular bone mineral density (Tb.BMD), tissue mineral density (Ct.TMD), tissue BMD (Ct.BMD), and trabecular bone separation (Tb.Sp) were measured.

### Bone proteomic analysis

The femurs of mice were cut into small pieces and then ground into powder by liquid nitrogen. SDT (4% SDS, 100 mM Tris–HCl, 1 mM DTT, pH 7.6) buffer was used for sample lysis and protein extraction. Filter-aided sample preparation (FASP) ultrafiltration method to remove descaling agents from protein samples. The samples were milled for three cycles at − 20 °C, 70 Hz, 60 s, with an interval of 30 s. The supernatant was carefully aspirated after centrifugation at 4 °C, 12,000 rpm for 15 min. The femur proteins of mice from the GD and control groups were identified and quantified using tandem mass tag (TMT)-labeled mass spectrometry [19, 30]. Differentially expressed proteins (DEPs) that were up and downregulated between the two groups were identified using the expression fold change (FC) criteria—FC > 1.3 or FC < 1/1.3 and *p*-value < 0.05 (*t*-test) [31]. The STRING website (https://string-db.org/) and Cytoscape software (version 3.7.1) were utilized to build the protein–protein interaction (PPI) network of DEPs based on the following criteria: (1) Mus musculus; (2) meaning of network edges as “confidence”; (3) minimum required interaction score as “medium confidence (0.400)”; (4) retaining the protein with adjusted *p* < 0.05; and (5) hiding the disconnected and interacted in pair nodes [32]. Gene Ontology (GO) and Kyoto Encyclopedia of Genes and Genomes (KEGG) enrichment and cluster analysis were used to screen candidate proteins or signaling pathways from DEPs [33, 34].

### Cell culture

MC3T3-E1 cells were purchased from Cyagen Biosciences Inc. (M7-0201, Guangzhou, China). Cells were cultured with Minimum Essential Medium Alpha (α-MEM, HyClone, Utah, USA) containing 10% fetal bovine serum (FBS) (BioInd, Israel), 100 U/mL streptomycin, and 100 U/mL penicillin (BioInd, Israel) in an incubator (Heal Force, HF-240) at 37 °C and 5% CO_2_. When the cell density reached 70–80%, it was digested with 0.25% trypsin (Yeasen) and then passaged.

### Cell viability assay

The viability of MC3T3-E1 cells was determined using a Cell Counting Kit (CCK-8) assay. Cells were seeded in 96-well plates (4 × 103 cells/well) overnight and then treated with triiodothyronine (T3, Sigma-Aldrich, St. Louis, USA) at 20, 50, and 100 nM concentrations for 3, 6, 12, 24, 48, and 72 h. Then, 10 μL of CCK-8 reagent (Solarbio, Beijing, China) per well was added and incubated for another 2 h. A Multiskan FC Microplate reader (Thermo Fisher Scientific, MA, USA) was used to measure the absorbance at an optical density of 450 nm.

### Quantitative real-time PCR

Total RNA was extracted using the Trizol (Invitrogen, California) technique. An equal amount of RNA was reverse transcribed into cDNA using the Hifair II 1st Strand cDNA Synthesis Kit (Yeasen, Shanghai, China). Moreover, quantitative real-time PCR (qRT-PCR) was conducted using a real-time fluorescence qPCR instrument (Applied Biosystems 7500, USA) and SYBR Green PCR Master Mix (Yeasen) according to the manufacturer's protocols. Relative gene expression was measured using the comparative CT method and normalized to the quantity of glyceraldehyde-3-phosphate dehydrogenase (GAPDH). The primer sequences (Takara, Tokyo, Japan) are listed in Table 1.

**Table 1:** Quantitative real-time PCR primer sequences

Genes	Forward primer (5′-3′)	Reverse primer (5′-3′)
Gapdh	TGTGTCCGTCGTGGATCTGA	TTGCTGTTGAAGTCGCAGGAG
Dap1	AAGCTGGAGACCAAAGCTGGAC	CGAGCAATAACGCCAGAGATGA
Cdkn1b	TCGACGCCAGACGTAAACAG	TCTCAGTGCTTATACAGGATGTCCA
Med14	CAGCTGTGGTCCTGAAATCCAA	GAATGTCTGCCTGCTGAGTGGTA
S100b	TCGGACACTGAAGCCAGAGA	GACATCAATGAGGGCAACCA
Cdk9	CAGCCTTACAGTCATGGAGTGACAA	TTAGGACCATCCCATCTCCACA
Lc3	CCCCAGTGGATTAGGCAGAG	CAGCCAGCACCCAAAAGAG
Becn1	CCAATGTCTTCAATGCCACCTTC	GGCAGCATTGATTTCATTCCAC
P62	GAAGCTGCCCTATACCCACATCTC	TGCTTCGAATACTGGATCGTGTC
Runx2	ATGCTTCATTCGCCTCACAAA	GCACTCACTGACTCGGTTGG
Ocn	CGCCTACAAACGCATCTACG	CAGAGAGAGAGGACAGGGAGGA

### Western blot analysis

Total protein was extracted from bone tissues and MC3T3-E1cells using radio immunoprecipitation assay (RIPA) lysis buffer (Solarbio) and quantified using a BCA protein assay kit (Beyotime, Jiangsu, China). Using sodium dodecyl sulfate polyacrylamide gel electrophoresis (SDS-PAGE), the protein samples were separated and then transferred to polyvinylidene fluoride (PVDF) membranes (Millipore, MA, USA), and blocked with 5% skimmed milk powder (Solarbio) for 1 h at room temperature. Subsequently, membranes were incubated with primary antibodies, including anti-DAP1 (Abcam Cat#ab32056, 1:10,000 dilution), anti-Beclin1 (Abcam Cat#ab207612, 1:2000 dilution), anti-LC3B (Abcam Cat#ab192890, 1:2000 dilution), anti-SQSTM1/p62 (Abcam Cat#ab109012, 1:10000 dilution), anti-RUNX family transcription factor 2 (RUNX2) (Huabio Cat#ET1612-47, 1:5000 dilution), anti- OCN (Abcam Cat#93876, 1:1500 dilution), anti-mammalian target of rapamycin (mTOR) (Abcam Cat#ab32028, 1:2000 dilution), anti-phospho-S2448 mTOR (p-mTOR) (Abcam Cat#ab109268, 1:2000 dilution), anti- ATG16L1 (Abmart Cat#T56909, 1:1000 dilution), anti-phospho-S278 ATG16L1 (p-ATG16L1) (Abcam Cat#ab195242, 1:1000 dilution), anti-WD repeat domain, phosphoinositide interacting 1 (WIPI1) (Abcam Cat#ab128901, 1:1000 dilution), anti-GAPDH (Immunoway Cat#YM3215, 1:5000 dilution) and anti-β-Tubulin (CST Cat#15115, 1:1000 dilution) overnight at 4 °C and with secondary antibody (Jackson, Pennsylvania, USA) for 1 h at room temperature. Finally, the protein bands were visualized using an enhanced chemiluminescence (ECL) kit (Bioscience, Shanghai, China) and quantified via an image analyzer (E-Blot, Hangzhou, China).

### Establishment of stable DAP1 overexpressing (OE) and KD MC3T3-E1 cell lines

To establish the DAP1-OE MC3T3-E1 cell line, the recombinant pLVX-mDap1-Puro plasmid (2 mg) was co-transfected with psPAX2 (1 mg) and pMD2.G (1 mg) into HEK293T cells in each well (six-well plate) to package the infectious lentivirus. MC3T3-E1 cells were infected with concentrated lentivirus (with 6 mg/mL of polybrene added simultaneously) for 24 h, followed by selection with 2 µg/mL puromycin.

The lentivirus vector expressed a small hairpin RNA (shRNA) against DAP1 mRNA or a control vector (GeneChem, Shanghai, China), which was then employed to create the MC3T3-E1 cell lines with stable KD of DAP1 and NC cells [35, 36]. MC3T3-E1 cells were seeded at a density of 2 × 10^4^/well in a 24-well plate and incubated overnight. Furthermore, the volume of lentiviruses was calculated at the multiplicity of infection (MOI = 70) according to the manufacturer's protocol, followed by its addition. The DAP1 OE and KD status of puromycin-resistant MC3T3-E1 mDap1 cells were validated with a DAP1 antibody.

### Transmission electron microscopy (TEM) assays

Autophagic vacuoles in MC3T3-E1 cells of the control, T3, DAP1-KD, DAP1-KD + 3-methyladenine (3-MA, 5 mM; Selleck, Houston, USA), and DAP1-OE groups were detected using TEM staining. The cell pellets were re-suspended and fixed with the TEM fixative (Servicebio, Cat#G1102) and then wrapped in 1% agarose solution after centrifugation. Sample-containing agarose blocks were shielded from light and fixed with 1% osmium tetroxide (Ted Pella Inc) in 0.1 M PBS (pH 7.4) for 2 h at room temperature. It was then dehydrated with graded alcohol and immersed in 100% acetone (Sinopharm, Cat#10000418) two times. Resin penetration and sample embedding were performed with a 1:1 ratio of acetone and EMBed 812 (SPI, Cat#90529-77-4) at 37 °C. The polymerized resin blocks were sliced to a 60–80 nm thickness on an ultramicrotome (Leica, UC7, Germany). The cuprum grids were stained with 2% uranium acetate saturated alcohol solution and protected from light for 8 min, followed by staining with 2.6% lead citrate and kept in a CO2-free environment for 8 min. After washing with ultrapure water and drying at room temperature overnight, the cuprum grids were examined and photographed under a TEM (HITACHI, HT7800/HT7700, Japan). Those with double or multiple membranes and cytoplasmic components were identified as initial autophagic vacuoles (AVi), and those with single membranes and degraded cytoplasmic components were categorized as degradative autophagic vacuoles (AVd) [37].

### Monitoring autophagic flux with tandem stubRFP-sensGFP-LC3 lentivirus

The tandem stubRFP-sensGFP-LC3 lentivirus (GeneChem, Shanghai, China) was used to detect autophagy flux in MC3T3-E1 cells according to the previously described protocol [38]. Tandem fluorescent LC3 puncta were evaluated 24 h after transfection of MC3T3-E1 cells with tandem stubRFP-sensGFP-LC3 lentivirus. Cells were washed thrice with PBS and fixed with 4% paraformaldehyde. Furthermore, images were obtained after DAPI (Solarbio) staining of the nuclei using confocal microscopy (Leica TCS SP8, Wetzlar, Germany).

### Immunohistochemistry assays

Mouse femur sections were rehydrated in a gradient of xylene and ethanol; then antigen retrieval was done with 1 mM Tris-Ethylene Diamine Tetraacetic Acid (EDTA, pH 9.0, Servicebio, Cat#G1203) for 20 min at 95 °C. Moreover, it was followed by the inactivation of endogenous peroxidase with 3% hydrogen peroxide solution (H2O2) for 25 min and blocking with 3% bovine serum albumin (BSA) for 1 h. Primary antibodies including anti-DAP1 (Abcam Cat#ab32056, 1:250 dilution), anti-LC3B (Abcam Cat#ab192890, 1:100 dilution), anti-RUNX2 (Huabio Cat#ET1612-47, 1:200 dilution), and anti-OCN (Abcam Cat#93876, 1:200 dilution) were incubated with sections overnight at 4 °C. After washing, sections were incubated with a secondary antibody for 1 h at room temperature. The sections were viewed, and images were taken using an optical microscope (Nikon Eclipse E100). The dark brown color denoted a positive expression. The positive area ratio of the positive pixel area to tissue pixel area was calculated to estimate the relative expression of the target proteins using Aipathwell software (Servicebio, Inc.)

### Masson staining

The relative area of collagen fibers, including newly formed bone tissue, was quantified in mouse femurs using Masson staining. Sections were dewaxed and hydrated in xylene and a series of graded alcohols. Subsequently, they were stained with Weigert’s hematoxylin for 10 min. Then, fully washed with water and differentiated with 1% hydrochloric acid alcohol. The sections were then stained with ponceau acid fuchsin solution for 10 min, washed with 1% glacial acetic acid for 10 s, differentiated with 1% phosphomolybdic acid aqueous solution for 5 min, and then stained directly with aniline blue for 5 min. Sections were rinsed with 1% glacial acetic acid, dehydrated with anhydrous ethanol, made transparent by xylene treatment, and sealed with neutral resin. Stained sections were observed under a light microscope (Nikon Eclipse E100, Japan), and images were captured. ImageJ software was utilized to compute the ratio of collagen fiber area to total cross-sectional area to estimate bone formation.

### PCR array analysis

Differentially expressed genes (DEGs) related to autophagy were discovered in T3(100 nM)-treated and DAP1-KD + T3(100 nM)-treated MC3T3-E1 for 24 h using an autophagy PCR array kit (Wcgene Biotech, Shanghai, China), including 90 candidate and 4 reference genes (Table 2). The RNA reverse transcription products (cDNA) and SYBR Green Master Mix were mixed and added into the 96-well plate for qRT-PCR in accordance with the manufacturer's protocol. The FC of the genes was calculated and log2-transformed; those with logFC > 1 were considered upregulated genes and those with logFC < 1 as downregulated genes. Subsequently, these DEGs were subjected to GO enrichment, KEGG pathway enrichment, and PPI network analysis using Metascape and the STRING website.

**Table 2:** Genes of autophagy PCR array kit

Gene classification (Mouse)	Genes
Autophagic vacuole formation	AMBRA1, ATG12, ATG5, ATG9B, BECN1, GABARAPL1, IRGM1, MAP1LC3A, MAP1LC3B, RGS19, ULK1, WIPI1, ATG4A
Transporter	ATG10, ATG16L1, ATG16L2, ATG3, ATG4A, ATG4B, ATG4C, ATG4D, ATG7, ATG9A, GABARAP, GABARAPL2, RAB24
Chaperone-mediated autophagy	HSP90AA1, HSPA8
Regulation of autophagy	AKT1, APP, BAD, BAK1, BAX, BCL2, BCL2L1, BID, BNIP3, CASP3, CASP8, CDKN1B, CDKN2A, CLN3, CTSB, CXCR4, DAPK1
Reference genes	ACTB, GAPDH, HPRT1, B2m

### Statistical analysis

GraphPad Prism 8.0 was used for statistical analysis. The data are presented as mean ± standard deviation (SD). A two-tailed Student's *t*-test was used for a single variable with a two-group comparison, whereas a one-way ANOVA was conducted in single-variable comparisons with more than two groups. The differences with *p* < 0.05 were considered statistically significant.

## Results

### Validation of mouse model of GD-induced OP

Following the Ad-TSHR289 immunization cycle, mice in the GD group (n = 8) exhibited a thin body with sparse fur (Additional file 1: Fig. S1B) and significantly lower body weight and serum TSH, accompanied by higher serum TT4 and TRAb, compared with the NC (n = 6) and control groups (n = 9) (Additional file 1: Fig. S1C). In GD mice, the distal femoral trabecular bone (Additional file 1: Fig. S1D) and bone microstructure parameters—Tb.BV/TV, Tb.Th, Tb.N, Tb.BMD, and Ct.BMD—were significantly reduced, whereas the Tb.Sp was significantly widened (Additional file 1: Fig. S1E). These findings confirmed a successful mouse model of GD-induced OP.

### Bone proteomic analysis

The principal component analysis (PCA) indicated good repeatability of protein samples from the same group of mouse femur origin (Additional file 2: Fig. S2A). Comparative proteomic analysis showed significant differential expression of proteins in the femoral tissues of control and GD mice (Additional file 2: Fig. S2B). A total of 4,489 quantifiable proteins were identified in the femur tissue of the control and GD mice, and 35 upregulated and 39 downregulated DEPs were recognized by differential expression analysis (Additional file 2: Fig. S2C). Subsequently, six signaling pathways were identified using the KEGG pathway analysis; DEPs were largely enriched in thyroid hormone and mTOR signaling pathways (Additional file 2: Fig. S2D). The PPI network predicted 51 nodes, 114 edges, and 4 functional clusters: supramolecular complex, stress response, blood coagulation, and PI3K-Akt-mTOR signaling pathway (Additional file 2: Fig. S2E).

### Validation of target protein DAP1

**Fig. 1:**
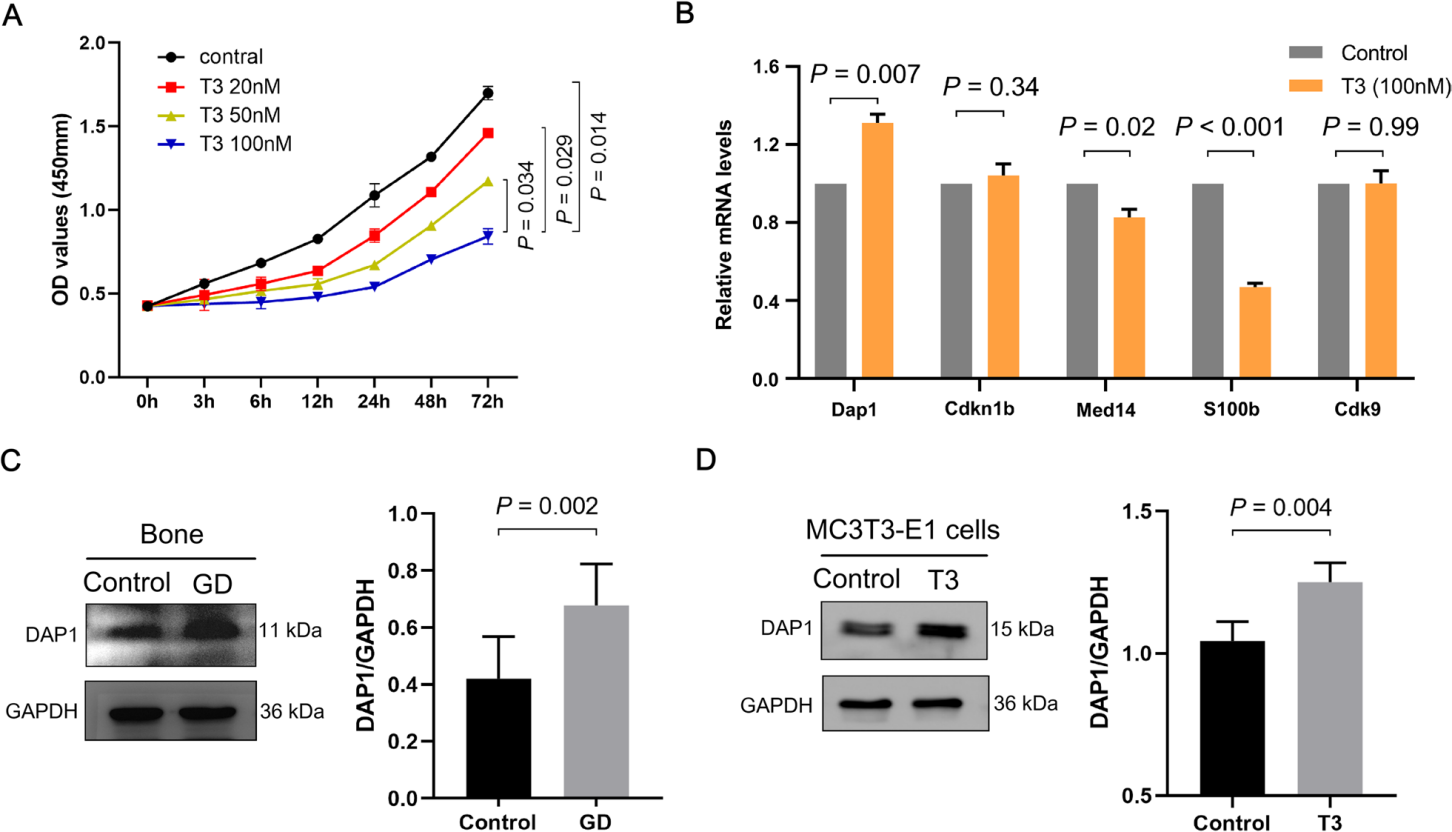
Screening and validation of target proteins. **A** The proliferation ability of MC3T3-E1 cells treated with various concentrations (20, 50, and 100 nM) of T3 for the indicated times. **B** Comparison of the relative expression levels and trends of the candidate genes in MC3T3-E1 cells treated with T3 (100 nM) for 24 h. **C** Representative images of DAP1 protein by western blotting of GD mouse bone tissue and **D** T3 (100 nM)-treated MC3T3-E1 cells for 24 h. Results represented as mean ± SD; n = 3

With Graves’ disease-induced osteoporosis and autophagy regulation as the research guide, the proteins obtained by overlap analysis of DEPs enriched in thyroid hormone signaling pathway, mTOR signaling pathway, and GO annotated as regulating apoptosis, autophagy, transcription, and oxidative stress were identified as candidate differential proteins. As a result, five potential proteins were screened, including four upregulated proteins: DAP1, mediator complex subunit 14 (MED14), S100 calcium-binding protein B (S100B), and cyclin-dependent kinase 9 (CDK9), and one downregulated protein: cyclin-dependent kinase inhibitor 1B (CDKN1B). Furthermore, T3 inhibited MC3T3-E1 cell viability in a concentration-dependent manner; significantly lower levels were found in the 100 nM group compared to the 20 and 50 nM groups and remained relatively stable over 24 h (Fig. 1A). The DAP1 mRNA relative expression level was significantly increased in T3-treated MC3T3-E1 cells compared to the control group. The expression trend of DAP1 was the only candidate protein consistent with the proteomics results (Fig. 1B). Similarly, DAP1 expression levels were significantly elevated in the bone tissues of GD mice (Fig. 1C) and T3-treated MC3T3-E1 cells (Fig. 1D). The target protein, DAP1, was highly expressed in bone tissue of GD mice and T3-treated osteoblasts.

### DAP1 KD in mouse bone promoted bone formation

**Fig. 2:**
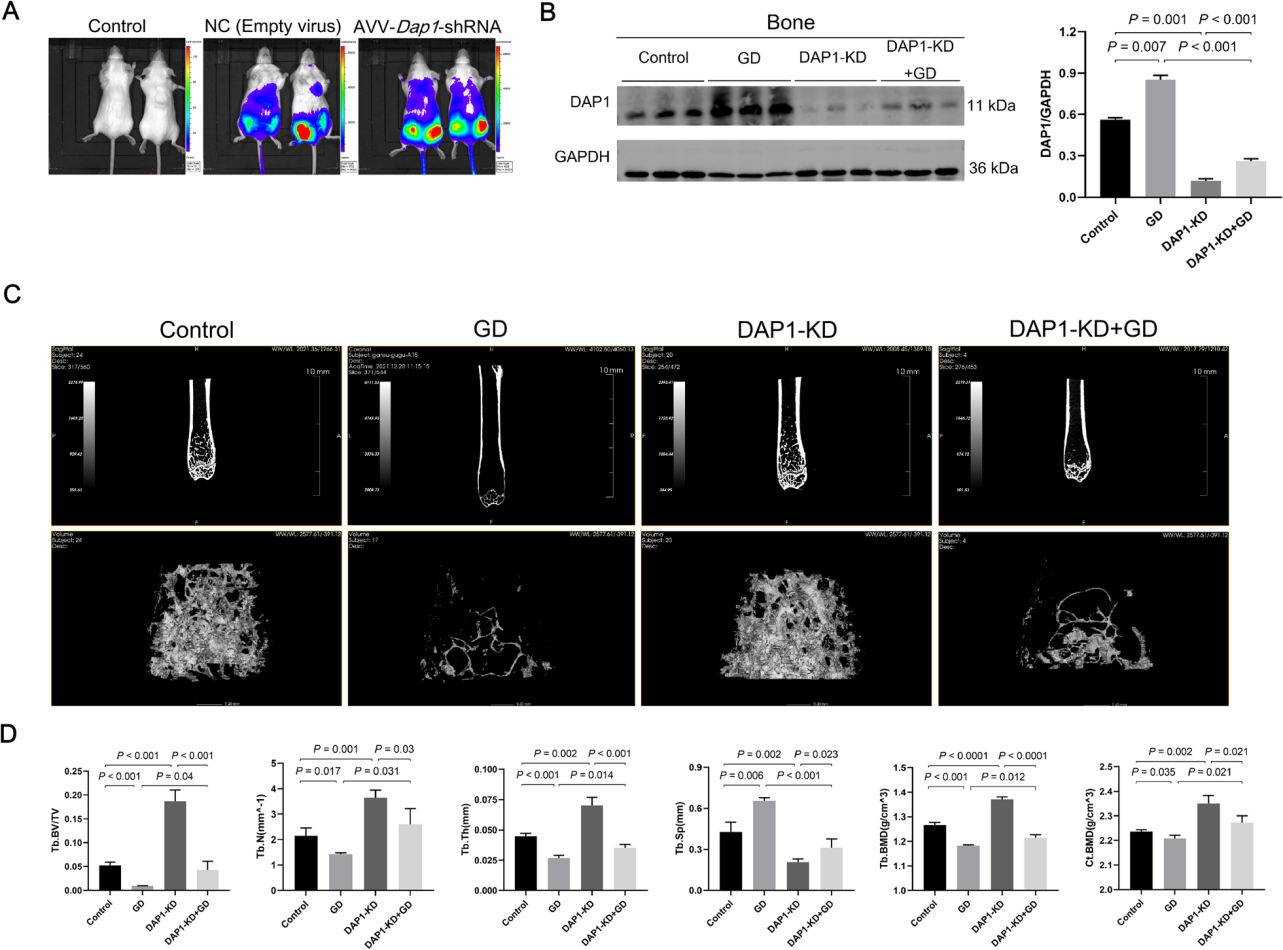
Validation of bone DAP1 knockdown (KD) in mice and its effect on bone microarchitecture. **A** Mouse live imaging of the effect of infection by an adeno-associated virus vector. **B** Representative immunoblots of the expression level of DAP1 protein in the femur. The bar graph shows a significantly reduced level of DAP1 protein expression in the femur of DAP1-KD mice and a significantly increased level in GD mice. Results represented as mean ± SD; n = 3. **C** Micro-CT images of the distal femoral metaphysis in the control, DAP1-KD, GD, and DAP1-KD + GD mice. The bone microarchitecture of DAP1-KD mice was significantly better than that of control mice. **D** The bar graphs depicting the Tb.BV/TV, Tb.Th, Tb.N, Tb.Sp, Tb.BMD, and Ct.BMD of the mouse femurs. Bone microarchitectural parameters were significantly improved in DAP1-KD + GD mice compared with those in GD mice. Results represented as mean ± SD. n = 3–4 per group

Live imaging of mice displayed luminescence saturation in the AAV9-Dap1-shRNA and AAV9-NC groups, particularly in the bilateral femurs (Fig. 2A). Western blot analysis of mouse femur protein showed that DAP1-KD mice (n = 6) had lower DAP1 expression levels, and GD mice (n = 8) had higher levels compared with control mice (n = 9), while DAP1-KD + GD mice (n = 7) had a restorative increase compared with DAP1-KD mice (Fig. 2B). The data confirmed successful KD of DAP1 in mouse bone by employing RNA interference technology. Micro-CT measurement of the distal femur of mice showed that DAP1-KD mice had the highest trabecular bone mass, whereas GD mice had the lowest, while DAP1-KD + GD mice displayed some improvement over GD mice (Fig. 2C). According to the bone microstructure analysis, Tb.BV/TV, Tb.N, Tb.Th, Tb.BMD, and Ct.BMD of mice femurs in the DAP1-KD group were significantly increased, while the Tb.Sp was significantly decreased, consistent with the performance of DAP1-KD + GD mice (Fig. 2D). In conclusion, DAP1 reduction in bone tissue promoted bone formation and enhanced bone microarchitecture in GD mice.

### DAP1 influences osteogenesis by negatively regulating autophagy in vitro and in vivo

**Fig. 3:**
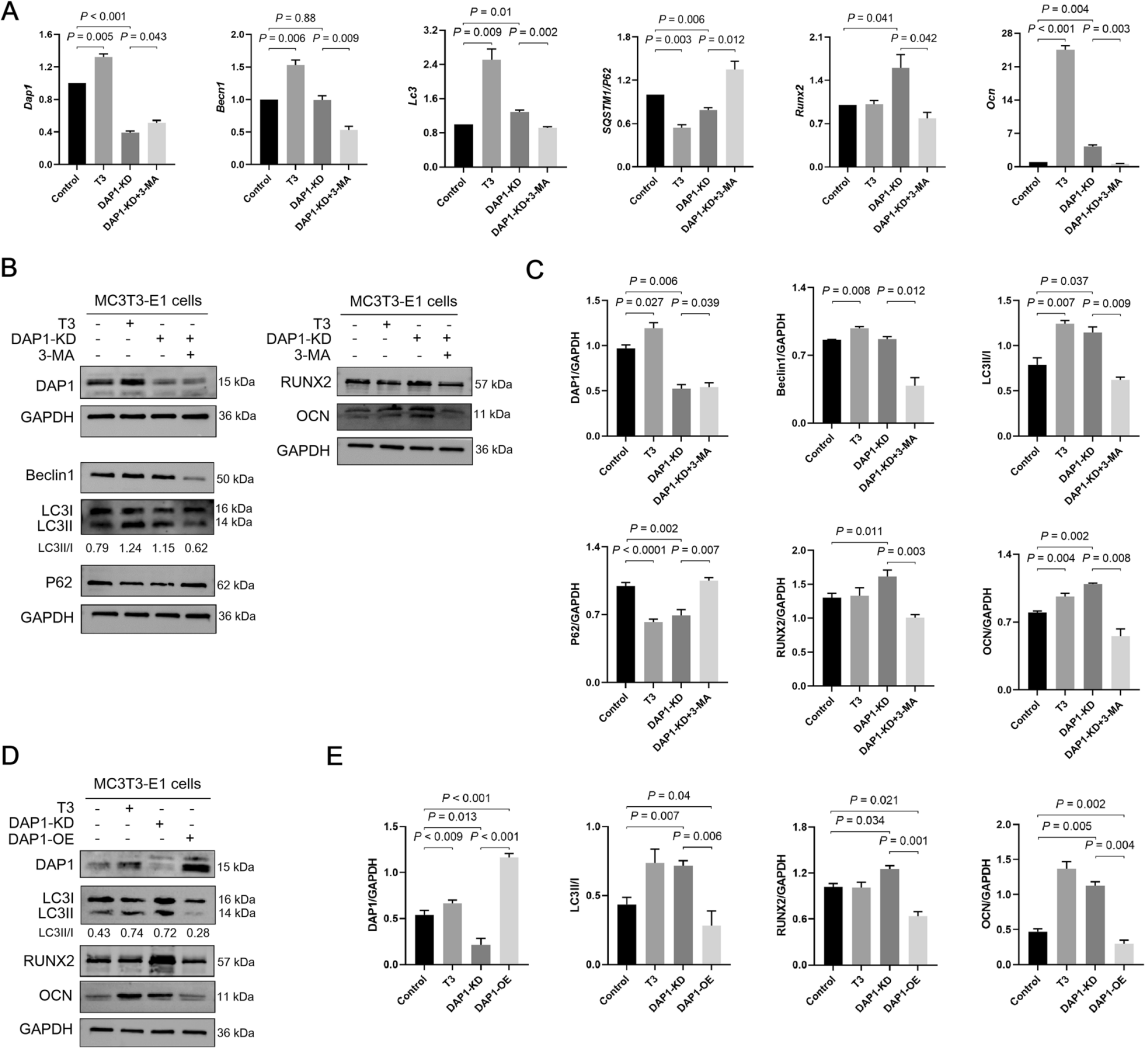
Effects of DAP1 on autophagy and osteogenic markers in MC3T3-E1 cells. **A** qRT-PCR analysis of the mRNAs level of Dap1, Becn1, Lc3, p62, Runx2, and Ocn in MC3T3-E1 cells in the control, T3 (100 nM treated for 24 h), DAP1-KD, and DAP1-KD + 3-MA (5 mM treated for 24 h) groups. **B** Representative images of DAP1, Beclin 1, LC3, p62, RUNX2, and OCN proteins by western blotting of MC3T3-E1 cells treated with T3 or/and 3-MA for 24 h in the above four groups. **C** The bar graphs display the expression level of autophagy markers Beclin 1, LC3, p62, and osteogenic markers RUNX2 and OCN in DAP1 knockdown cells treated with T3 or/and 3-MA for 24 h. **D** Representative images of DAP1, LC3, RUNX2, and OCN proteins by western blotting of MC3T3-E1 cells in the control, T3, DAP1-KD, and DAP1-OE groups. **E** The bar graphs display the expression level of LC3, RUNX2, and OCN. Results are represented as mean ± SD; n = 3

It has been previously demonstrated that T3 increases DAP1 expression in MC3T3-E1 cells. Therefore, we utilized the changes in DAP1 to detect the expression levels of autophagy markers Beclin1, LC3II/I ratio, p62 and osteogenic markers RUNX2, OCN from control, T3 (100 nM)-treated for 24 h, and DAP1-KD MC3T3-E1 cells. Meanwhile, DAP1-KD cells treated with 3-MA (5 mM) for 24 h, an autophagy inhibitor, were used to further confirm the effect of DAP1 on autophagy and osteogenesis. DAP1 mRNA and protein expression levels were significantly lower in MC3T3-E1 cells of DAP1-KD and higher in T3-treated cells. Moreover, T3-treated cells had higher levels of Beclin 1, while it remained unchanged in DAP1-KD cells. Cells treated with T3 and DAP1-KD showed a significant increase in the LC3II/I ratio and a decrease in p62. DAP1-KD increased RUNX2 and OCN in the cells, while T3 only increased OCN. However, these autophagy markers in DAP1-KD cells could be inhibited by 3-MA; RUNX2 and OCN were significantly inhibited (Fig. 3A–C). Furthermore, the LC3II/I ratio, RUNX2, and OCN were significantly lower in DAP1-OE cells than in DAP1-KD cells (Fig. 3D–E). These results indicated that DAP1 negatively regulated LC3 lipidation (LC3I transforms into LC3II) and osteogenesis in MC3T3-E1 cells.

**Fig. 4:**
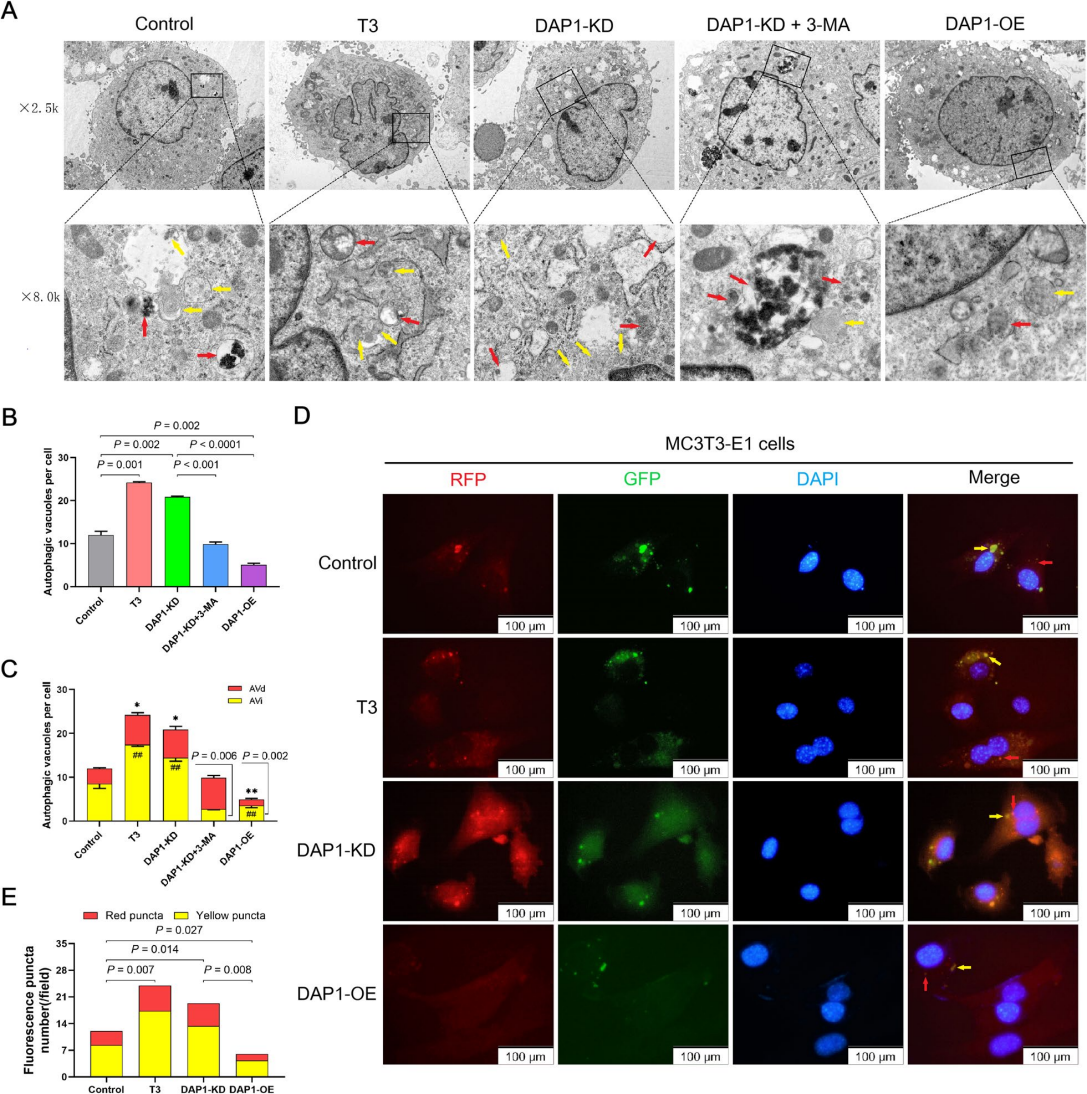
Effects of DAP1 on autophagic vacuoles and flux in MC3T3-E1 cells. **A** Representative transmission electron microscopy (TEM) images of autophagic vacuoles in MC3T3-E1 cells in the control, T3 (100 nM treated for 24 h), DAP1-KD, DAP1-KD + 3-MA (5 mM treated for 24 h), and DAP1-OE groups. Yellow arrowheads point to the initial autophagic vacuoles (AVi), and red arrowheads point to the degradative autophagic vacuoles (AVd). Magnification: 5000 and 8000 times. **B** The bar graphs display the average number of autophagic vacuoles per cell treated with T3 (100 nM) or/and 3-MA (5 mM) for 24 h in each group and **C** the number of AVi and AVd per cell in each group. At least three to five cells were counted in each group in three different fields. Results are represented as mean ± SEM; n = 3. **D** Representative fluorescent microscopy images of LC3 fluorescence puncta in MC3T3-E1 cells in the control, T3 (100 nM treated for 24 h), DAP1-KD and DAP1-OE groups. Yellow fluorescent puncta represent autophagosomes, and red fluorescent puncta represent autophagolysosomes. Nuclei were stained with DAPI (blue). Scale bar: 100 μm. (E) Quantification of LC3 puncta with the RFP (red) fluorescence relative to the total (yellow) signal in each field. Result are represented as mean ± SD; n = 3. *p < 0.05, **/##*p* < 0.01

We observed the autophagic vacuoles in MC3T3-E1 cells by TEM to differentiate among AVi and AVd (Fig. 4A). When compared to the control group, cells in the T3-treated and DAP1-KD groups had a significantly higher number of autophagic vacuoles, whereas it was significantly decreased in cells of the DAP1-KD group following the application of 3-MA. In contrast, significantly fewer autophagic vacuoles were present in cells of the DAP1-OE group compared to the DAP1-KD group (Fig. 4B). AVi and AVd were significantly increased in the DAP1-KD group than the control group, whereas 3-MA significantly reduced them. Compared to the DAP1-KD cells, DAP1-OE cells had significantly reduced AVi and AVd (Fig. 4C). To examine the influence of DAP1 on autophagic flux, we infected MC3T3-E1 cells with a stubRFP-sensGFP-LC3 lentiviral system. DAP1-KD cells had increased yellow (for autophagosomes) and red (for autophagolysosomes) fluorescent puncta than control cells, with relatively more yellow fluorescent puncta, suggesting that reduction of DAP1 promotes autophagic flux via encouraging autophagosomes production. In contrast, both fluorescent puncta were diminished in cells exhibiting DAP1 overexpression, particularly the yellow fluorescent puncta; DAP1 overexpression decreased autophagic flux by inhibiting autophagosomes formation (Fig. 4D). Autophagic flux was significantly increased in T3-treated and DAP1-KD cells and decreased in DAP1-OE cells (Fig. 4E). These findings suggest that DAP1 inhibited autophagic vacuole formation and autophagic flux in MC3T3-E1 cells.

**Fig. 5:**
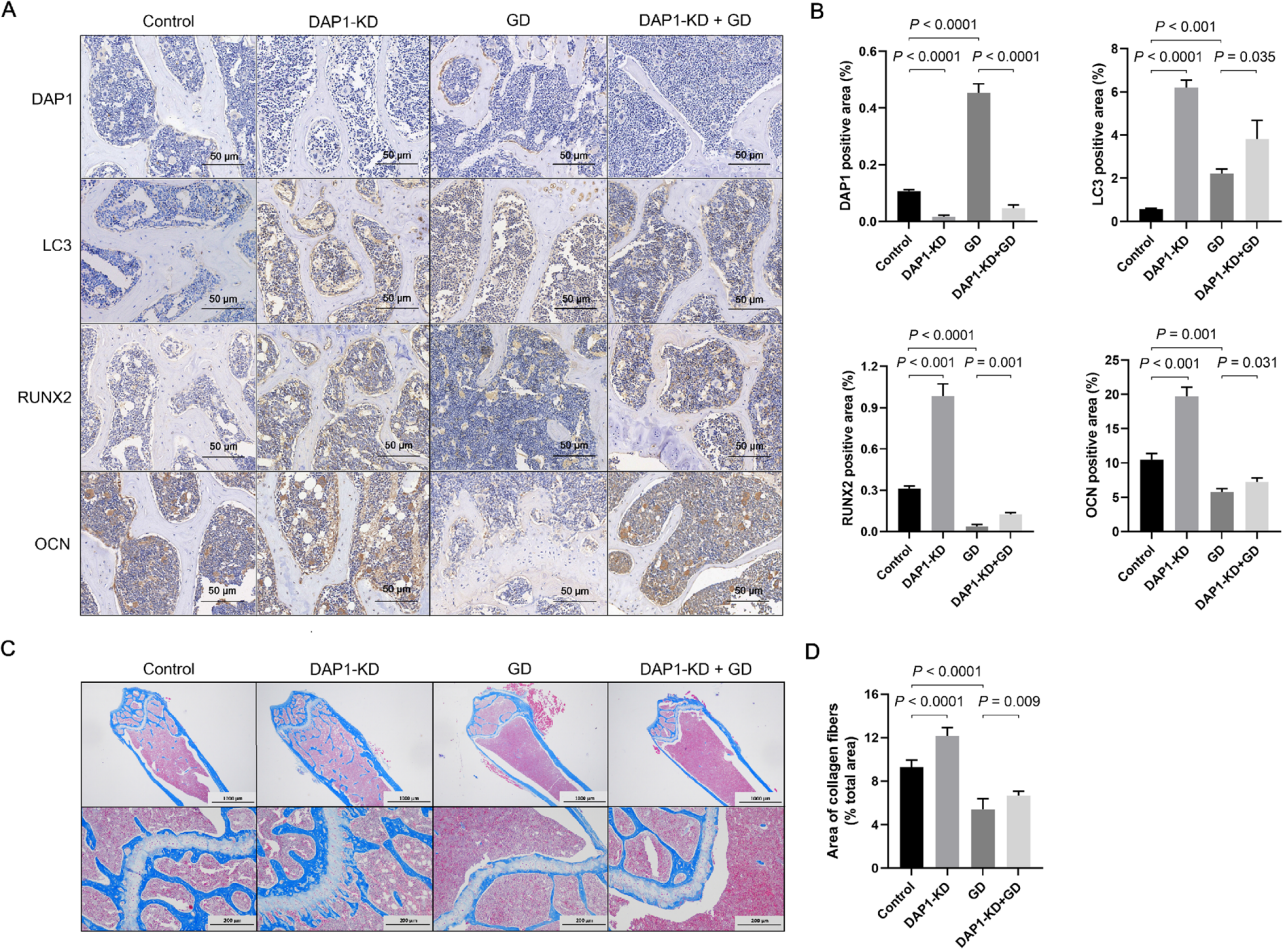
Immunohistochemistry of autophagy and osteogenic markers and Masson staining of collagen fibers in mouse femurs of the indicated groups. **A** Representative immunohistochemistry-stained images of DAP1, LC3, RUNX2, and OCN proteins in the distal femoral sections of each group (shown in dark brown in images). Scale bar = 50 μm. **B** The bar graph displays the relative positive expression area of DAP1, LC3, RUNX2, and OCN in each group (n = 6/group). The femurs of DAP1-KD mice showed lower levels of DAP1 and higher levels of LC3, RUNX2, and OCN. **C** Representative Masson-stained images of the distal femur in each group. The collagen fibers were stained blue, and the muscle fibers, cellulose and red blood cells were stained red. Scale bar = 1000 and 200 μm. **D** The bar graphs display the relative area of collagen fibers in each group (n = 6/group). Results represented as mean ± SD; n = 6

We examined the expression levels of LC3, RUNX2, and OCN by immunohistochemistry in the bones of control, DAP1-KD, GD, and DAP1-KD + GD mice to reveal the effects of DAP1 on autophagy and osteogenesis. The results revealed that DAP1 expression was significantly lower in the femurs of DAP1-KD mice and higher in GD mice. DAP1-KD mice had significantly higher levels of LC3, RUNX2, and OCN expression than control mice, and DAP1-KD + GD mice had significantly higher levels than GD mice. As a result, DAP1 deficiency in mouse bone promoted autophagy and osteogenesis (Fig. 5A–B). Masson staining revealed that DAP1-KD mice had significantly more stained area and GD mice had significantly less stained area when compared to control mice. GD mice with DAP1-KD, on the other hand, showed a restorative increase when compared to GD mice. These results demonstrate that decreased expression of DAP1 promotes collagen fiber production in bone (Fig. 5C–D).

### Autophagy-related qPCR array analysis identified Mtor/Atg16L1/Wipi1 as a potential effector molecular of DAP1-regulated autophagy

The PCR arrays identified 20 downregulated and 26 upregulated autophagy-related DEGs from the control and DAP1-KD MC3T3-E1 cells treated with T3 (100 nM) for 24 h (Additional file 3: Fig. S3A). GO enrichment analysis was performed using Metascape to predict the function of target genes associated with DAP1 through three aspects using Metascape: biological process (BP), cell composition (CC), and molecular function (MF). The BP mainly included autophagy, macroautophagy, autophagosome assembly and apoptotic process. The CC analysis mainly included autophagosome, autophagosome membrane, vacuole, vacuolar membrane, phagophore assembly site. MF analysis largely enriched in the binding of enzymes, proteins, proteases, ubiquitin-protein ligases, and kinases. Thus, GO enrichment analysis demonstrated that DAP1 may be involved in the formation of autophagosomes in MC3T3-E1 cells in response to T3, which in turn regulates the cellular autophagic process (Additional file 3: Fig. S3B).The autophagy and mitophagy in animals, various other autophagy, apoptosis, and the P53 signaling pathway were significantly enriched in the KEGG pathway enrichment analysis. These results confirmed that DAP1 plays a biological role in MC3T3-E1 cells through autophagy and apoptosis signaling pathways (Additional file 3: Fig. S3C). PPI analysis constructed three functional clusters: regulation of autophagy containing seven autophagy-regulated genes, autophagic vesicle formation containing eight autophagic vesicle-forming genes, and transporters containing nine transporter genes. Among these genes, Becn1 (Beclin 1), Mtor, and Casp3 (caspase 3) ranked the top three with node degrees of 34, 33, and 27, respectively. It can be seen that the protein interactions of DEGs are mainly focused on autophagosome formation and regulation (Additional file 3: Fig. S3D). Furthermore, overlap analysis of DEGs enriched in the autophagy signaling pathway and the GO enrichment analysis revealed that Atg16L1, Ulk1, Wipi1, and Becn1 may be involved in the DAP1-mediated autophagy signaling pathway (Additional file 3: Fig. S3E).According to our findings, there was no difference in Beclin1 level between DAP1-KD and control cells. Finally, we identified *Mtor*, *Atg16L1*, and *Wipi1* as potential key genes involved in DAP1-regulated autophagy in T3-treated MC3T3-E1 cells.

### DAP1 regulates autophagy in MC3T3-E1 cells via the ATG16L1–LC3 axis

**Fig. 6:**
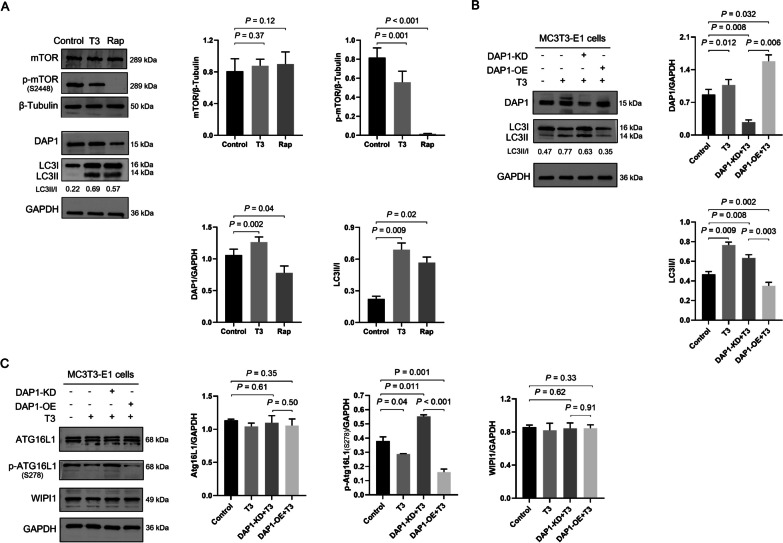
DAP1 regulates autophagy in MC3T3-E1 cells via the ATG16L1–LC3 axis. **A** Representative images of mTOR, p-mTOR (Ser2448), DAP1, and LC3 proteins of MC3T3-E1 by western blotting in control, T3 (100 nM treated for 24 h), and rapamycin (Rap, 200 mM treated for 24 h) groups. The bar graphs showed a significant decrease in p-mTOR and an increase in LC3II/I ratio in the T3 and Rap groups; however, the expression of DAP1 protein was inconsistent. **B** Representative images of DAP1 and LC3 proteins of MC3T3-E1 cells by western blotting in the control, T3, DAP1-KD + T3, and DAP1-OE + T3 groups after 24 h. The bar graph shows that DAP1 expression was inversely proportional to the LC3II/I ratio. **C** Representative images of ATG16L1, p-ATG16L1 (Ser278), and WIPI1 proteins of MC3T3-E1 cells by western blotting from the indicated groups after 24 h. The bar graphs shows that p-ATG16L1 was significantly increased in the DAP1-KD + T3 group and decreased in the T3 and DAP1-OE + T3 groups, and there was no difference in WIPI1 among the groups. Results represented as mean ± SD; n = 3

Rap, a specific mTOR inhibitor, was used to inhibit the phosphorylation of mTOR in MC3T3-E1 cells, thereby activating autophagy. Then p-mTOR, DAP1 expression levels and LC3 II/I ratio were compared in control, T3 and Rap-treated cells to demonstrate whether T3 affects the autophagy regulatory role of DAP1 through mTOR. The T3 and Rap-treated groups showed a significant decrease in p-mTOR and a significant increase in LC3 II/I ratio compared to the control group. However, DAP1 increased in the T3 group, while it was significantly decreased in the Rap group (Fig. 6A). Thus, mTOR may not be unnecessary for DAP1-mediated regulation of autophagy in T3-treated MC3T3-E1 cells. Subsequently, we assessed p-ATG16L1 and WIPI1 expressions. There was no significant difference between groups in the expression of total ATG16L1. In contrast, p-ATG16L1 was highly expressed in the DAP1-KD + T3 group, which was followed by an increase in the LC3II/I ratio, whereas it was lowly expressed in the T3 and DAP1-OE + T3 groups, which was followed by a decrease in the LC3II/I ratio. Furthermore, T3 and DAP1 had no effect on WIPI1 expression (Fig. 6B–C). These findings demonstrated that DAP1-regulated LC3 lipidation is mediated by p-ATG16L1. Finally, DAP1 regulates autophagy in MC3T3-E1 cells via the ATG16L1–LC3 axis.

## Discussion

GD-induced OP involves the osteoblast autophagic process [17, 18]; the regulatory mechanism is complex and largely undefined [9]. Understanding the autophagic regulatory mechanism of osteoblasts is critical for successfully treating GD-induced OP. The most common cause of hyperthyroidism is GD, which is characterized by elevated thyroid hormone levels that have a significant impact on bone homeostasis [14]; therefore, we used T3 as an extrinsic stimulus for the cellular model. We discovered a novel regulatory molecule, DAP1, in GD-induced OP via bone proteomic analysis. DAP1 is a critical protein that negatively regulates autophagy [20], and while it has been studied in numerous tumor cells [22, 23] and lymphocytes of the DAP1 haplotype [39], no evidence exists with respect to osteoblasts. In addition, we confirmed that DAP1 negatively regulated autophagy and osteogenesis in GD-induced OP and osteoblasts by knocking down DAP1 in mouse bone and MC3T3-E1 cells. DAP1 regulated autophagy in MC3T3-E1 cells primarily during the autophagic vacuole formation phase, according to the findings of the TEM assay and the LC3 dual fluorescence indicator system. PCR array analysis, DAP1 overexpression, and KD cell models all revealed the molecular mechanism by which T3 regulates autophagy in osteoblasts at the same time.

Proteomics analysis has been widely used in innovative research [40, 41], but bone proteomics has received less attention [19]. Furthermore, we used Ad-TSHR289 immunoinjection to perform tandem mass tag (TMT) proteomics on femurs from normal and GD-induced OP mice. A sufficient number of quantifiable proteins were discovered using quantitative proteomics techniques. The validation of candidate genes in T3-treated MC3T3-E1 cells revealed that the increasing trend of Dap1 was consistent with the bone proteomics findings. In addition, in vivo and in vitro targeting validation revealed that DAP1 protein levels were significantly higher in GD mouse femurs and T3-treated MC3T3-E1 cells. Thus, we determined that DAP1 may be involved in the GD-induced OP process, with a focus on T3 affecting osteoblasts via DAP1. Previous research discovered that DAP1 mediates gamma interferon-induced apoptosis in HeLa and other tumor cells [42, 43]. In 2010, DAP1 was found to have a negative regulatory effect on autophagy when HeLa cells were repeatedly stimulated to apoptosis [20]. Preliminary evidence suggests that low DAP1 expression induces autophagy in cells, although the underlying regulatory mechanism is unknown [22, 23]. Koren et al. [44] demonstrated that DAP1 KD boosted LC3 lipidation and autophagosome accumulation. DAP1 KD increased the LC3II/I ratio (also known as LC3 lipidation) and decreased p62 in MC3T3-E1 cells, but 3-MA, an inhibitor of autophagic vacuole formation, mitigated these changes. As a result, the role of DAP1 in osteoblast autophagy has been established. Furthermore, TEM and LC3 fluorescent double-labeled lentiviral systems revealed a significant increase in autophagic vacuoles and a relative increase in AVi in DAP1 KD MC3T3-E1 cells, whereas DAP1 OE cells showed decreased total autophagic vacuoles and AVi. These findings strongly suggest that reducing DAP1 increases autophagic flux in osteoblasts, promoting the formation of autophagic vacuoles. Thus, DAP1 regulates the LC3II/I ratio to form autophagic vacuoles in osteoblasts. Meanwhile, RUNX2 and OCN levels were significantly higher in DAP1 KD MC3T3-E1 cells that were inhibited by 3-MA; DAP1 overexpression inhibited RUNX2 and OCN expression. This suggests that the negative regulation of autophagy by DAP1 has a significant impact on osteogenesis in osteoblasts. Micro-CT and Masson staining demonstrated enhanced bone formation in DAP1 KD mice in vivo. DAP1 KD GD mice had better bone microarchitecture and collagen fibers than GD mice. Immunohistochemistry of mouse femurs revealed increased expression of LC3, RUNX2, and OCN after knockdown of DAP1 in normal control or GD mice. These results revealed that DAP1 has a negative correlation with autophagy and osteogenesis in osteoblasts and bone. Autophagy, as a dynamic recycling system, is involved in cell and tissue renewal [3]. Osteoblasts may be the target cells of DAP1 to regulate autophagy in bone. As a result, DAP1 may be an important therapeutic target for GD-induced OP.

According to our findings, DAP1 overexpression inhibits LC3 lipidation in osteoblasts, while DAP1 KD encourages LC3 lipidation. However, the molecular mechanism behind this regulation process in osteoblasts is unknown and has not been reported in other cell types [23, 45]. Finally, cellular autophagy signaling pathway PCR arrays identified four key genes associated with the biological function of DAP1. Although T3 and Rap inhibited p-mTOR and promoted autophagy [46], DAP1 expression was variable. As a result, mTOR is not involved in T3-regulated DAP1 expression. However, DAP1 could be an mTOR substrate for regulating autophagy in osteoblasts, consistent with the findings of Koren et al. [20] Despite an increase in LC3 lipidation following DAP1 KD, Beclin-1 remained unchanged. Beclin-1, unlike T3, may not be required by DAP1 to regulate autophagy in osteoblasts [47]. WIPI1, another target protein, interacts with ATG16L1 at the membrane to bind with phosphatidylinositol and promote autophagosome formation [48]; however, we found no change in WIPI1, which could be attributed to its inability to regulate autophagy in T3-treated MC3T3-E1 cells, unlike WIPI2, which strongly binds to ATG16L1 and promotes autophagy in HEK293 cells [49]. Multiple autophagy inducers have been demonstrated to phosphorylate ATG16L1 as a conserved signaling pathway for autophagy activation [50]. DAP1 was discovered to significantly regulate the phosphorylation level of ATG16L1 in a negative correlated manner. DAP1 overexpression reduced p-ATG16L1, inhibiting LC3 lipidation, whereas DAP1 knockdown had the opposite effect. Furthermore, ATG16L1 interacts with ATG12-ATG5 to form a complex that mediates PEbinding to LC3 (MAP1LC3A, MAP1LC3B, or MAP1LC3C), resulting in recognition and catalysis of the LC3 lipidation site to produce the membrane-bound activated form of LC3II, and promotes autophagosomal membrane elongation [51–53]. DAP1 KD MC3T3-E1 cells had significantly more autophagic vacuoles than DAP1 OE cells. Tian et al. suggested that p-ATG16L1 was present only on newly formed autophagosomes [50]. Similarly, in response to T3, DAP1 inhibits ATG16L1 phosphorylation at serine (S278), affecting LC3 lipidation and thus regulating neoautophagosome formation. The role of DAP1 in mediating autophagy regulation by TSH and TRAb during GD needs to be investigated further. Finally, DAP1 mediates T3-mediated autophagy regulation in MC3T3-E1 cells via the ATG16L1–LC3 axis.

Negative autophagy regulation in bone and osteoblasts is simply a functional study of DAP1 in the GD state. Increased DAP1 may not always be harmful in GD-induced OP. Autophagy was found to be increased in the bone tissue of GD mice and T3-treated MC3T3-E1 cells, which was consistent with previous findings [17, 54, 55], DAP1 negatively regulates autophagy; thus, why is it increased in bones and cells but not decreased? Thyroid hormone and GD induce autophagy through a variety of mechanisms; for example, T3 promotes autophagy in osteoblasts via endoplasmic reticulum stress [56] and reactive oxygen species [47]. The regulation of autophagy during GD-induced OP by thyroid hormone, TSH and TRAb, as well as the autophagic changes in osteoblasts, osteoclasts and bone marrow mesenchymal stem cells, are more complex [9, 57, 58]. As a result, we hypothesize that DAP1 not only regulates autophagy in GD mouse bone tissue and T3-treated MC3T3-E1 cells, but also inhibits overactivated autophagy as an autophagic homeostatic mechanism. Koren et al. [44] referred to this mechanism of DAP1 as an autophagic “brake” model.

## Conclusion

In summary, DAP1 inhibits LC3 lipidation in osteoblasts via the ATG16L1–LC3 axis, influencing the formation of new autophagosomes. Furthermore, increased DAP1 levels in bone tissue from GD-induced OP and T3-treated osteoblasts may be a key mechanism in regulating autophagic homeostasis.

### Supplementary Information

**Additional file 1**. **Figure S1**: Establishment of mouse models for Graves' disease (GD)-induced osteoporosis (OP). (A) Immune cycle schedule of mice injected with Ad-TSHR289. (B) Representative mouse body shape in the control, NC, and GD groups at the end of the immune cycle. The GD mice showed significant weight loss and sparse fur. (C) Comparisons of the mouse weight, serum TSH, TT4, and TRAb. (D) Representative three-dimensional reconstruction images of the mouse distal femur detected by micro-CT. (E) Comparison of microstructural parameters of trabecular bone, including Tb.BV/TV, Tb.N, Tb.Th, Tb.Sp, Tb.BMD, and Ct.BMD. Results represented as mean ± SD.

**Additional file 2**. **Figure S2**: Bioinformatics analysis of bone proteomics. (A) The protein quantitative principal component analysis (PCA) diagram of all samples. (B)Heat map of shared protein levels in two mouse femoral sources. (C)The volcanogram of differential proteins in the femur of control and GD mice. Red plots represent upregulated proteins, blue represent downregulated proteins, and gray plots represent indifferent proteins. (D) Six signaling pathways obtained by KEGG pathway enrichment analysis of DEPs. The circle size indicates the number of DEPs in the functional class or pathway and the circle color indicates the enrichment significance p-value. (E) Protein-protein interaction (PPI) network of differentially expressed proteins identified by mouse bone proteomics. The circles display the 4 functional clusters.

**Additional file 3**. **Figure S3**: Bioinformatics analysis of potential key genes mediating the DAP1-regulated autophagy signaling pathway by PCR array analysis in MC3T3-E1 cells of the control and T3 (100 nM treated for 24 h) groups. (A) The 20 downregulated and 26 upregulated differentially expressed genes (DEGs) were identified by the Log2-FC value cut-off at 1. (B) GO enrichment analysis of DEGs from BP, CC, and MF. (C) The top 20 pathways obtained from KEGG enrichment analysis of DEGs. (D) The PPI of DEGs included three well-defined functional clusters. Red represents upregulated genes and blue represents downregulated genes. (E) Venn diagram of the overlap between the enriched genes of autophagy pathway and GO analysis. Four co-overlapping genes may be involved in mediating the DAP1-regulated autophagy pathway.

## Data Availability

The datasets used and/or analyzed during the current study are available from the corresponding author on reasonable request.

## References

[1] Mizushima N, Levine B, Cuervo AM, Klionsky DJ (2008). Autophagy fights disease through cellular self-digestion. Nature.

[2] Liang JR, Lingeman E, Luong T, Ahmed S, Muhar M, Nguyen T, Olzmann JA, Corn JE (2020). A Genome-wide ER-phagy screen highlights key roles of mitochondrial metabolism and ER-resident UFMylation. Cell.

[3] Mizushima N, Komatsu M (2011). Autophagy: renovation of cells and tissues. Cell.

[4] Russell RC, Tian Y, Yuan H, Park HW, Chang YY, Kim J, Kim H, Neufeld TP, Dillin A, Guan KL (2013). ULK1 induces autophagy by phosphorylating Beclin-1 and activating VPS34 lipid kinase. Nat Cell Biol.

[5] Pyo JO, Nah J, Jung YK (2012). Molecules and their functions in autophagy. Exp Mol Med.

[6] Ichimura Y, Kumanomidou T, Sou YS, Mizushima T, Ezaki J, Ueno T, Kominami E, Yamane T, Tanaka K, Komatsu M (2008). Structural basis for sorting mechanism of p62 in selective autophagy. J Biol Chem.

[7] Mizushima N, Levine B (2010). Autophagy in mammalian development and differentiation. Nat Cell Biol.

[8] Collier JJ, Guissart C, Oláhová M, Sasorith S, Piron-Prunier F, Suomi F, Zhang D, Martinez-Lopez N, Leboucq N, Bahr A, Azzarello-Burri S, Reich S, Schöls L, Polvikoski TM, Meyer P, Larrieu L, Schaefer AM, Alsaif HS, Alyamani S, Zuchner S, Barbosa IA, Deshpande C, Pyle A, Rauch A, Synofzik M, Alkuraya FS, Rivier F, Ryten M, McFarland R, Delahodde A, McWilliams TG, Koenig M, Taylor RW (2021). Developmental consequences of defective ATG7-mediated autophagy in humans. N Engl J Med.

[9] Yin X, Zhou C, Li J, Liu R, Shi B, Yuan Q, Zou S (2019). Autophagy in bone homeostasis and the onset of osteoporosis. Bone Res.

[10] Li X, Xu JK, Dai BY, Wang XL, Guo QY, Qin L (2020). Targeting autophagy in osteoporosis: from pathophysiology to potential therapy. Age Res Rev.

[11] Nollet M, Santucci-Darmanin S, Breuil V, Al-Sahlanee R, Cros C, Topi M, Momier D, Samson M, Pagnotta S, Cailleteau L, Battaglia S, Farlay D, Dacquin R, Barois N, Jurdic P, Boivin G, Heymann D, Lafont F, Lu SS, Dempster DW, Carle GF, Pierrefite-Carle V (2014). Autophagy in osteoblasts is involved in mineralization and bone homeostasis. Autophagy.

[12] Ignaszak-Szczepaniak M, Horst-Sikorska W, Dytfeld J, Gowin E, Słomski R, Stajgis M (2011). Association between estrogen receptor alpha gene polymorphisms and bone mineral density in Polish female patients with Graves’ disease. Acta Biochim Pol.

[13] Yi HS, Kim JM, Ju SH, Lee Y, Kim HJ, Kim KS (2016). Multiple fractures in patient with Graves’ disease accompanied by isolated hypogonadotropic hypogonadism. J Bone Metab.

[14] Kim HY, Mohan S (2013). Role and mechanisms of actions of thyroid hormone on the skeletal development. Bone Res.

[15] Bours SP, van Geel TA, Geusens PP, Janssen MJ, Janzing HM, Hoffland GA, Willems PC, van den Bergh JP (2011). Contributors to secondary osteoporosis and metabolic bone diseases in patients presenting with a clinical fracture. J Clin Endocrinol Metab.

[16] Nicholls JJ, Brassill MJ, Williams GR, Bassett JH (2012). The skeletal consequences of thyrotoxicosis. J Endocrinol.

[17] Yi L, Zhong T, Huang Y, Huang S (2020). Triiodothyronine promotes the osteoblast formation by activating autophagy. Biophys Chem.

[18] Huang B, Wang Y, Wang W, Chen J, Lai P, Liu Z, Yan B, Xu S, Zhang Z, Zeng C, Rong L, Liu B, Cai D, Jin D, Bai X (2015). mTORC1 prevents preosteoblast differentiation through the notch signaling pathway. PLoS Genet.

[19] Mickleburgh HL, Schwalbe EC, Bonicelli A, Mizukami H, Sellitto F, Starace S, Wescott DJ, Carter DO, Procopio N (2021). Human bone proteomes before and after decomposition: investigating the effects of biological variation and taphonomic alteration on bone protein profiles and the implications for forensic proteomics. J Proteome Res.

[20] Koren I, Reem E, Kimchi A (2010). DAP1, a novel substrate of mTOR, negatively regulates autophagy. Curr Biol: CB.

[21] Yahiro K, Tsutsuki H, Ogura K, Nagasawa S, Moss J, Noda M (2014). DAP1, a negative regulator of autophagy, controls SubAB-mediated apoptosis and autophagy. Infect Immun.

[22] Wazir U, Khanzada ZS, Jiang WG, Sharma AK, Kasem A, Mokbel K (2014). The interaction between DAP1 and autophagy in the context of human carcinogenesis. Anticancer Res.

[23] Nie X, Chen H, Niu P, Zhu Y, Zhou J, Jiang L, Li D, Lin M, Chen Z, Shi D (2020). DAP1 negatively regulates autophagy induced by cardamonin in SKOV3 cells. Cell Biol Int.

[24] Holthoff HP, Goebel S, Li Z, Fassbender J, Reimann A, Zeibig S, Lohse MJ, Munch G, Ungerer M (2015). Prolonged TSH receptor A subunit immunization of female mice leads to a long-term model of Graves’ disease, tachycardia, and cardiac hypertrophy. Endocrinology.

[25] Diana T, Holthoff HP, Fassbender J, Wüster C, Kanitz M, Kahaly GJ, Ungerer M (2020). A novel long-term Graves’ disease animal model confirmed by functional thyrotropin receptor antibodies. Eur Thyroid J.

[26] Wang W, Wang B, Sun S, Cao S, Zhai X, Zhang C, Zhang Q, Yuan Q, Sun Y, Xue M, Ma J, Xu F, Wei S, Chen Y (2021). Inhibition of adenosine kinase attenuates myocardial ischaemia/reperfusion injury. J Cell Mol Med.

[27] Nakamura-Takahashi A, Tanase T, Matsunaga S, Shintani S, Abe S, Nitahara-Kasahara Y, Watanabe A, Hirai Y, Okada T, Yamaguchi A, Kasahara M (2020). High-level expression of alkaline phosphatase by adeno-associated virus vector ameliorates pathological bone structure in a hypophosphatasia mouse model. Calcif Tissue Int.

[28] Adams ST, Mofford DM, Reddy GS, Miller SC (2016). Firefly luciferase mutants allow substrate-selective bioluminescence imaging in the mouse brain. Angew Chem (Int Engl).

[29] Percie du Sert N, Hurst V, Ahluwalia A, Alam S, Avey MT, Baker M, Browne WJ, Clark A, Cuthill IC, Dirnagl U, Emerson M, Garner P, Holgate ST, Howells DW, Karp NA, Lazic SE, Lidster K, MacCallum CJ, Macleod M, Pearl EJ, Petersen OH, Rawle F, Reynolds P, Rooney K, Sena ES, Silberberg SD, Steckler T, Würbel H (2020). The ARRIVE guidelines 2.0: updated guidelines for reporting animal research. PLoS Biol.

[30] Muraoka S, Jedrychowski MP, Yanamandra K, Ikezu S, Gygi SP, Ikezu T (2020). Proteomic profiling of extracellular vesicles derived from cerebrospinal fluid of Alzheimer’s disease patients: a pilot study. Cells.

[31] Lyu YS, Shao YJ, Yang ZT, Liu JX (2020). Quantitative proteomic analysis of ER stress response reveals both common and specific features in two contrasting ecotypes of arabidopsis thaliana. Int J Mol Sci.

[32] Chen Y, Li H, Lai L, Feng Q, Shen J (2020). Identification of common differentially expressed genes and potential therapeutic targets in ulcerative colitis and rheumatoid arthritis. Front Genet.

[33] Li Z, Jiang C, Li X, Wu WKK, Chen X, Zhu S, Ye C, Chan MTV, Qian W (2018). Circulating microRNA signature of steroid-induced osteonecrosis of the femoral head. Cell Prolif.

[34] Sakunrangsit N, Pholtaisong J, Sucharitakul J, Wanna-Udom S, Prombutara P, Pisitkun P, Leelahavanichkul A, Aporntewan C, Greenblatt MB, Lotinun S (2021). Identification of candidate regulators of mandibular bone loss in FcγRIIB(-/-) Mice. Sci Rep.

[35] Ferreira MV, Cabral ET, Coroadinha AS (2021). Progress and perspectives in the development of lentiviral vector producer cells. Biotechnol J.

[36] Chen Q, Shi F, Yang C, Mao G, Zhou C, Liu L, Yang X, Song Y (2021). Lentivirus-shRNA mediated prolyl hydroxylase 2 knockdown increases HIF-1α and inhibits nucleus pulposus cells degeneration. Cells Tissues Organ.

[37] Martins-Marques T, Catarino S, Zuzarte M, Marques C, Matafome P, Pereira P, Girão H (2015). Ischaemia-induced autophagy leads to degradation of gap junction protein connexin43 in cardiomyocytes. Biochem J.

[38] Lei Y, Xu X, Liu H, Chen L, Zhou H, Jiang J, Yang Y, Wu B (2021). HBx induces hepatocellular carcinogenesis through ARRB1-mediated autophagy to drive the G(1)/S cycle. Autophagy.

[39] Raj P, Song R, Zhu H, Riediger L, Jun DJ, Liang C, Arana C, Zhang B, Gao Y, Wakeland BE, Dozmorov I, Zhou J, Kelly JA, Lauwerys BR, Guthridge JM, Olsen NJ, Nath SK, Pasare C, van Oers N, Gilkeson G, Tsao BP, Gaffney PM, Gregersen PK, James JA, Zuo X, Karp DR, Li QZ, Wakeland EK (2020). Deep sequencing reveals a DAP1 regulatory haplotype that potentiates autoimmunity in systemic lupus erythematosus. Genome Biol.

[40] Frese CK, Mikhaylova M, Stucchi R, Gautier V, Liu Q, Mohammed S, Heck AJR, Altelaar AFM, Hoogenraad CC (2017). Quantitative map of proteome dynamics during neuronal differentiation. Cell Rep.

[41] Arbelaiz A, Azkargorta M, Krawczyk M, Santos-Laso A, Lapitz A, Perugorria MJ, Erice O, Gonzalez E, Jimenez-Agüero R, Lacasta A, Ibarra C, Sanchez-Campos A, Jimeno JP, Lammert F, Milkiewicz P, Marzioni M, Macias RIR, Marin JJG, Patel T, Gores GJ, Martinez I, Elortza F, Falcon-Perez JM, Bujanda L, Banales JM (2017). Serum extracellular vesicles contain protein biomarkers for primary sclerosing cholangitis and cholangiocarcinoma. Hepatology.

[42] Levy-Strumpf N, Kimchi A (1998). Death associated proteins (DAPs): from gene identification to the analysis of their apoptotic and tumor suppressive functions. Oncogene.

[43] Wybranska I, Polus A, Mikolajczyk M, Knapp A, Sliwa A, Zapala B, Staszel T, Dembinska-Kiec A (2013). Apoptosis-related gene expression in glioblastoma (LN-18) and medulloblastoma (Daoy) cell lines. Hum Cell.

[44] Koren I, Reem E, Kimchi A (2010). Autophagy gets a brake: DAP1, a novel mTOR substrate, is activated to suppress the autophagic process. Autophagy.

[45] Wiedemann C, Voigt J, Jirschitzka J, Häfner S, Ohlenschläger O, Bordusa F (2021). Backbone and nearly complete side-chain chemical shift assignments of the human death-associated protein 1 (DAP1). Biomol NMR Assign.

[46] Yau WW, Singh BK, Lesmana R, Zhou J, Sinha RA, Wong KA, Wu Y, Bay BH, Sugii S, Sun L, Yen PM (2019). Thyroid hormone (T(3)) stimulates brown adipose tissue activation via mitochondrial biogenesis and MTOR-mediated mitophagy. Autophagy.

[47] Sinha RA, Singh BK, Zhou J, Wu Y, Farah BL, Ohba K, Lesmana R, Gooding J, Bay BH, Yen PM (2015). Thyroid hormone induction of mitochondrial activity is coupled to mitophagy via ROS-AMPK-ULK1 signaling. Autophagy.

[48] De Leo MG, Berger P, Mayer A (2021). WIPI1 promotes fission of endosomal transport carriers and formation of autophagosomes through distinct mechanisms. Autophagy.

[49] Dooley HC, Razi M, Polson HE, Girardin SE, Wilson MI, Tooze SA (2014). WIPI2 links LC3 conjugation with PI3P, autophagosome formation, and pathogen clearance by recruiting Atg12-5-16L1. Mol Cell.

[50] Tian W, Alsaadi R, Guo Z, Kalinina A, Carrier M, Tremblay ME, Lacoste B, Lagace D, Russell RC (2020). An antibody for analysis of autophagy induction. Nat Methods.

[51] Mizushima N, Kuma A, Kobayashi Y, Yamamoto A, Matsubae M, Takao T, Natsume T, Ohsumi Y, Yoshimori T (2003). Mouse Apg16L, a novel WD-repeat protein, targets to the autophagic isolation membrane with the Apg12-Apg5 conjugate. J Cell Sci.

[52] Fujita N, Itoh T, Omori H, Fukuda M, Noda T, Yoshimori T (2008). The Atg16L complex specifies the site of LC3 lipidation for membrane biogenesis in autophagy. Mol Biol Cell.

[53] Gammoh N (2020). The multifaceted functions of ATG16L1 in autophagy and related processes. J Cell Sci.

[54] Mizuguchi Y, Morimoto S, Kimura S, Takano N, Yamashita K, Seki Y, Bokuda K, Yatabe M, Yatabe J, Watanabe D, Ando T, Ichihara A (2018). Prediction of response to medical therapy by serum soluble (pro)renin receptor levels in Graves' disease. PLoS ONE.

[55] Yao QM, Zhu YF, Wang W, Song ZY, Shao XQ, Li L, Song RH, An XF, Qin Q, Li Q, Zhang JA (2018). Polymorphisms in autophagy-related gene IRGM are associated with susceptibility to autoimmune thyroid diseases. Biomed Res Int.

[56] Li H, Li D, Ma Z, Qian Z, Kang X, Jin X, Li F, Wang X, Chen Q, Sun H, Wu S (2018). Defective autophagy in osteoblasts induces endoplasmic reticulum stress and causes remarkable bone loss. Autophagy.

[57] Morshed S, Latif R, Davies TF (2022). Rescue of thyroid cells from antibody induced cell death via induction of autophagy. J Autoimmun.

[58] Qiang Z, Jin B, Peng Y, Zhang Y, Wang J, Chen C, Wang X, Liu F (2019). miR-762 modulates thyroxine-induced cardiomyocyte hypertrophy by inhibiting Beclin-1. Endocrine.

