# Supplementary figures and images for: DAP1 regulates osteoblast autophagy via the ATG16L1–LC3 axis in Graves’ disease-induced osteoporosis
Source: J Orthop Surg Res. 2023 Sep 21;18:711. doi: 10.1186/s13018-023-04171-z (PMC10512661; doi:10.1186/s13018-023-04171-z)

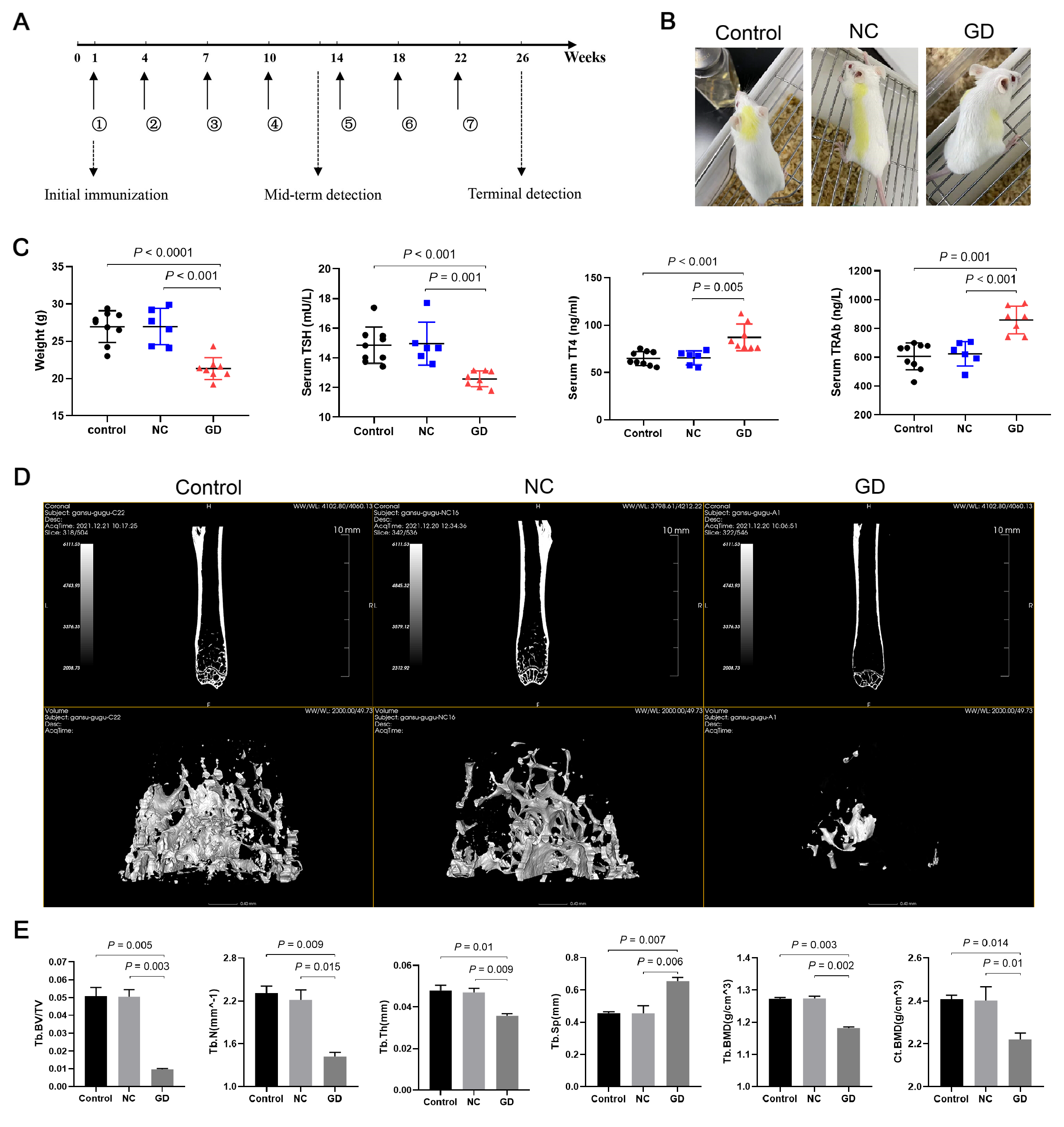

Supplement: Supplementary file 1 — Additional file 1. Figure S1: Establishment of mouse models for Graves' disease (GD)-induced osteoporosis (OP). (A) Immune cycle schedule of mice injected with Ad-TSHR289. (B) Representative mouse body shape in the control, NC, and GD groups at the end of the immune cycle. The GD mice showed significant weight loss and sparse fur. (C) Comparisons of the mouse weight, serum TSH, TT4, and TRAb. (D) Representative three-dimensional reconstruction images of the mouse distal femur detected by micro-CT. (E) Comparison of microstructural parameters of trabecular bone, including Tb.BV/TV, Tb.N, Tb.Th, Tb.Sp, Tb.BMD, and Ct.BMD. Results represented as mean ± SD. [file 13018_2023_4171_MOESM1_ESM.tiff]

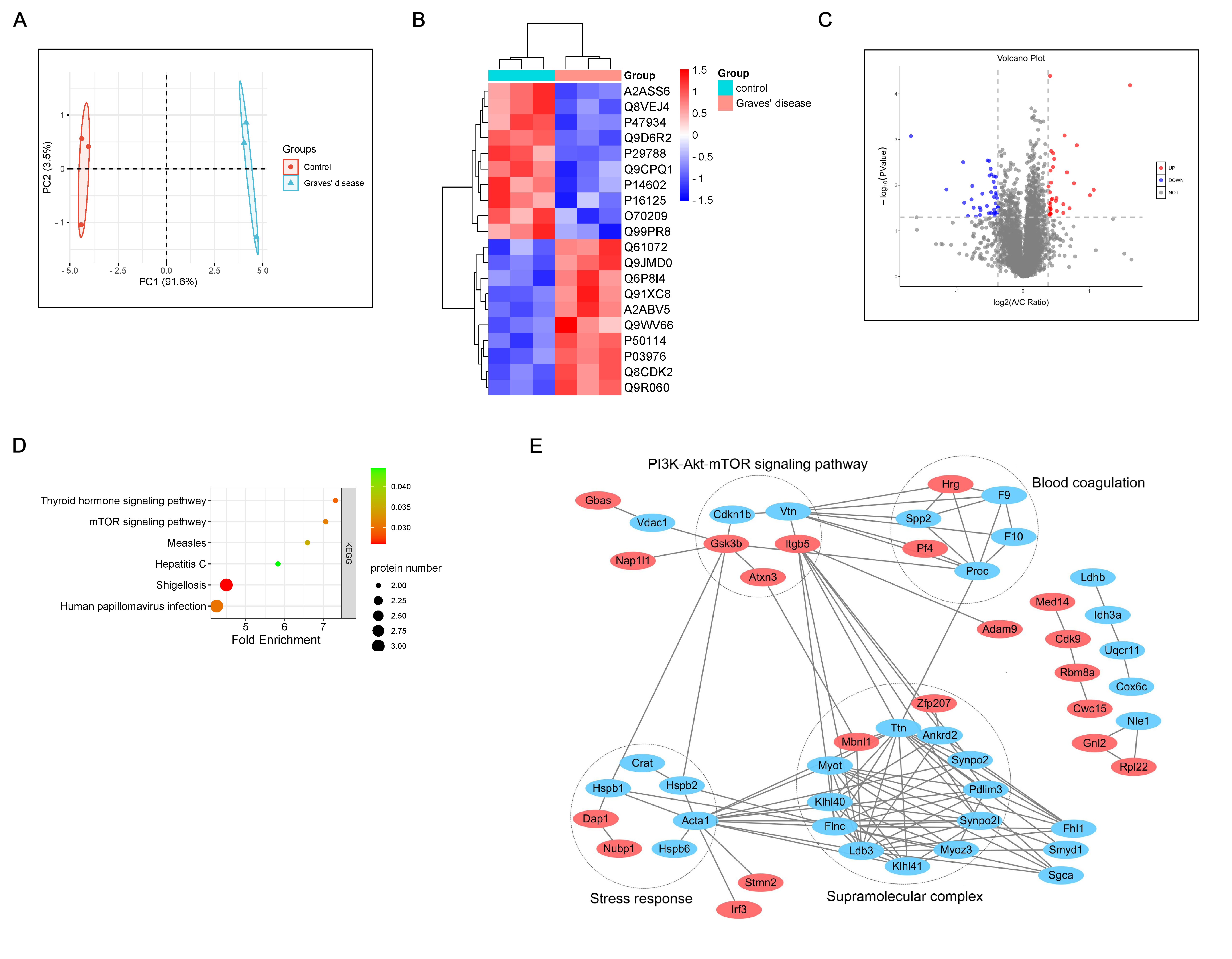

Supplement: Supplementary file 2 — Additional file 2. Figure S2: Bioinformatics analysis of bone proteomics. (A) The protein quantitative principal component analysis (PCA) diagram of all samples. (B)Heat map of shared protein levels in two mouse femoral sources. (C)The volcanogram of differential proteins in the femur of control and GD mice. Red plots represent upregulated proteins, blue represent downregulated proteins, and gray plots represent indifferent proteins. (D) Six signaling pathways obtained by KEGG pathway enrichment analysis of DEPs. The circle size indicates the number of DEPs in the functional class or pathway and the circle color indicates the enrichment significance p-value. (E) Protein-protein interaction (PPI) network of differentially expressed proteins identified by mouse bone proteomics. The circles display the 4 functional clusters. [file 13018_2023_4171_MOESM2_ESM.png]

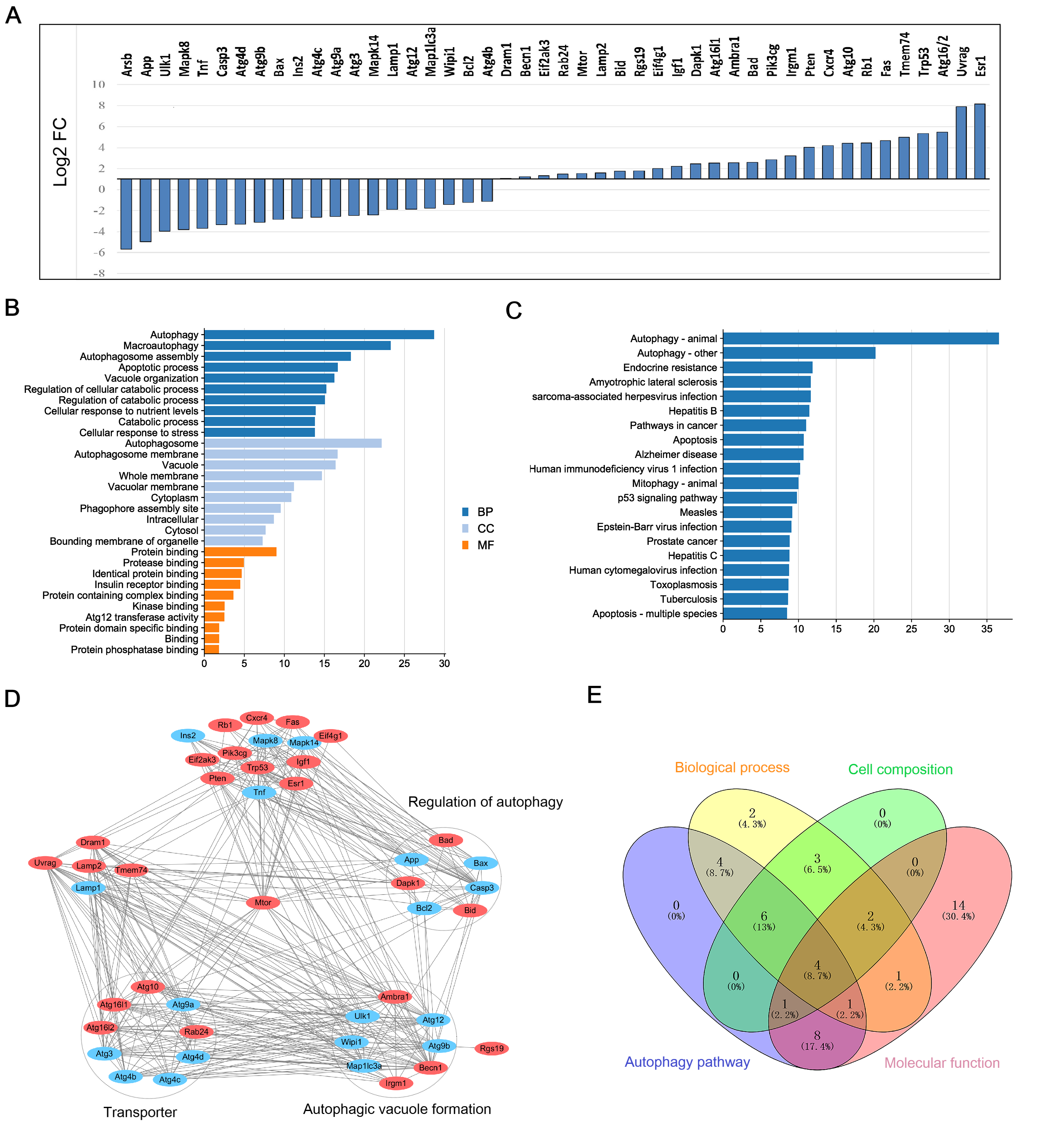

Supplement: Supplementary file 3 — Additional file 3. Figure S3: Bioinformatics analysis of potential key genes mediating the DAP1-regulated autophagy signaling pathway by PCR array analysis in MC3T3-E1 cells of the control and T3 (100 nM treated for 24 h) groups. (A) The 20 downregulated and 26 upregulated differentially expressed genes (DEGs) were identified by the Log2-FC value cut-off at 1. (B) GO enrichment analysis of DEGs from BP, CC, and MF. (C) The top 20 pathways obtained from KEGG enrichment analysis of DEGs. (D) The PPI of DEGs included three well-defined functional clusters. Red represents upregulated genes and blue represents downregulated genes. (E) Venn diagram of the overlap between the enriched genes of autophagy pathway and GO analysis. Four co-overlapping genes may be involved in mediating the DAP1-regulated autophagy pathway. [file 13018_2023_4171_MOESM3_ESM.tiff]
